# Development and interpretable validation of a machine-learning model for edentulism risk in US adults: A cross-sectional study integrating five algorithms and dietary data from NHANES 2009–2014

**DOI:** 10.1097/MD.0000000000051182

**Published:** 2026-10-30

**Authors:** Shiyuan Wu, Muze Huang, Zi-han Shen, Yaw-Syan Fu, Rong Wang

**Affiliations:** a Department of Blood Transfusion, Affiliated Hospital of Youjiang Medical University for Nationalities, Baise, China; b School of Stomatology, Xiamen Medical College, Xiamen, Fujian, China; c School of Basic Medical Science, Fujian Medical University, Fuzhou, Fujian, China; d School of Clinical Medicine, Xiamen Medical College, Xiamen, Fujian, China; e School of Basic Medical Science, Xiamen Medical College, Xiamen, Fujian, China; f Guangxi Key Laboratory of Artificial Intelligence for Genetic Diseases of Long-dwelling Nationalities, Baise, Guangxi, China.

**Keywords:** dietary factors, edentulism, machine learning, NHANES, risk prediction model, SHAP analysis

## Abstract

Edentulism is an end-stage oral condition that severely impairs the quality of life and overall health of older adults. Using cross-sectional data from the National Health and Nutrition Examination Survey (NHANES), this study integrated multidimensional information – including sociodemographic factors, chronic disease history, and dietary characteristics – to develop and validate an interpretable machine learning model for accurate prediction of edentulism risk. A retrospective cross-sectional design was adopted, including 9230 NHANES participants aged ≥ 20 years from 2005 to 2018, among whom the prevalence of edentulism was 7.86%. Participants were split into training and testing sets using a 70%:30% stratified random allocation. LASSO regression was first applied to select 11 core variables from 44 candidate features: age, years of education, smoking status, poverty-to-income ratio (PIR), magnesium, dietary fiber, total sugar, caffeine, diabetes history, and heart disease history. Five machine learning models (Random Forest, XGBoost, LightGBM, Decision Tree, and KNN) were then constructed, and their performance in the testing set was comprehensively evaluated based on discrimination (AUC, AUPRC), calibration (ECE), and clinical net benefit (decision curve analysis [DCA]). SHAP (SHapley Additive exPlanations) analysis was subsequently performed to interpret the optimal model. Baseline characteristics indicated that individuals with edentulism exhibited a pattern of “advanced age – low socioeconomic status – insufficient dietary antioxidant intake – accumulation of chronic conditions.” Restricted cubic splines (RCS) showed significant nonlinear protective associations of dietary fiber and magnesium intake with edentulism risk, whereas total sugar intake demonstrated a nonlinear positive association. Among all models, gradient boosting algorithms (XGBoost, LightGBM) achieved the highest discrimination (AUC = 0.855). Considering discrimination, reliability of predicted probabilities (ECE = 0.0097), and clinical net benefit, Random Forest was ultimately selected as the optimal model. SHAP analysis revealed that age, years of education, smoking status, and PIR were the most influential predictors of edentulism risk. This study successfully developed a Random Forest–based prediction model for edentulism risk, demonstrating strong discriminative performance, reliable calibration, and substantial clinical net benefit. Age, socioeconomic factors, and smoking status were identified as core risk determinants, while inadequate magnesium and dietary fiber intake emerged as key nutritional intervention targets. The model provides a useful decision-support tool for early screening and precision nutrition strategies among high-risk populations.

## 1. Introduction

Against the backdrop of accelerating global population aging, oral health has become a key indicator of healthy aging.^[1]^ Edentulism, the end stage of progressive periodontitis and dental caries, not only leads to impaired mastication and compromised nutritional intake but is also strongly associated with cardiovascular diseases, cognitive decline, and increased all-cause mortality.^[2-4]^ It is therefore recognized as a “terminal oral condition” that significantly diminishes the quality of life among older adults. Although the prevalence of tooth loss has declined in some developed countries due to advances in preventive medicine, edentulism remains disproportionately common among individuals with lower socioeconomic status and a high burden of comorbidities.^[5-7]^ Consequently, early and precise identification of high-risk individuals is of great public health importance for halting disease progression and advancing the front line of chronic oral disease prevention.

Most previous studies on edentulism risk prediction have relied on traditional epidemiologic approaches, predominantly using linear models such as logistic regression to evaluate the effects of classical risk factors including age, smoking, and socioeconomic status.^[8,9]^ However, high-impact research in recent years has highlighted that the human body is a highly complex biological system, where disease development often arises from high-dimensional nonlinear interactions among environmental exposures, behaviors, and biological markers rather than simple linear accumulation.^[10,11]^ Traditional linear models face inherent limitations in addressing multicollinearity and in capturing complex nonlinear threshold effects between variables (e.g., “U-shaped” or “L-shaped” nutrient–risk curves), which may lead to a ceiling effect in predictive performance and restrict their potential for individualized clinical decision-making.

Among the modifiable risk factors, dietary nutrient intake – particularly antioxidant nutrients – has attracted increasing scholarly attention. Oxidative stress is considered a central pathological mechanism underlying periodontal tissue destruction and alveolar bone loss.^[12,13]^ Epidemiologic evidence suggests that adequate intake of magnesium, dietary fiber, and antioxidant vitamins (e.g., vitamins C and D) may help mitigate inflammatory cascades and slow the progression of tooth loss.^[14-16]^ However, existing evidence largely stems from analyses focusing on single nutrients and lacks comprehensive predictive models that simultaneously integrate multiple dietary components with systemic chronic disease characteristics after accounting for confounding. Furthermore, few studies have investigated whether these nutrients exhibit specific dose–response thresholds in relation to edentulism risk, limiting the establishment of clear target values for nutrition-based precision interventions.

With the rapid development of artificial intelligence, machine learning (ML) algorithms have emerged as a new paradigm for disease risk prediction due to their powerful capacity for data mining and modeling nonlinear relationships. Compared with traditional statistical methods, ensemble algorithms such as Random Forest, extreme gradient boosting (XGBoost), and light gradient boosting machine (LightGBM) can more effectively capture complex interactions in high-dimensional datasets and substantially improve predictive performance.^[17,18]^ However, such advanced ML models often face the challenge of being “black boxes,” where high predictive accuracy comes at the expense of interpretability, making it difficult for clinicians to understand the underlying decision logic and hindering their adoption in clinical practice.

To address these knowledge gaps, this study leveraged nationally representative cross-sectional data from the National Health and Nutrition Examination Survey (NHANES 2005–2018). We integrated multidimensional features – including sociodemographic factors, chronic disease history, and dietary antioxidant intake – to construct and compare 5 machine learning models, including Random Forest and XGBoost. Beyond developing a highly accurate prediction tool for edentulism risk, we utilized SHAP (SHapley Additive exPlanations) to provide post hoc interpretability based on game-theoretic principles, enabling intuitive visualization of the direction and magnitude of key features (e.g., dietary fiber and magnesium intake) in individual risk prediction. Our goal was to develop a model that combines strong discriminative ability with clinical interpretability, thereby offering scientific support for high-risk population screening and precision nutrition strategies in the prevention of edentulism.

## 2. Materials and methods

### 2.1. Study population and data source

This retrospective cross-sectional study utilized data from the National Health and Nutrition Examination Survey (NHANES) 2005–2018, a continuous, nationally representative survey designed to evaluate the health and nutritional status of the noninstitutionalized US population. All NHANES protocols received ethical approval, and written informed consent was obtained from all participants.

Individuals aged ≥20 years were initially screened. Edentulism, the primary outcome, was defined as complete loss of natural dentition and coded as a binary variable (0 = non-edentulous; 1 = edentulous). Participants with missing information on the outcome or any key predictor were excluded to ensure analytical completeness and model robustness. The final sample selection process is presented in Figure 1.

**Figure 1.**
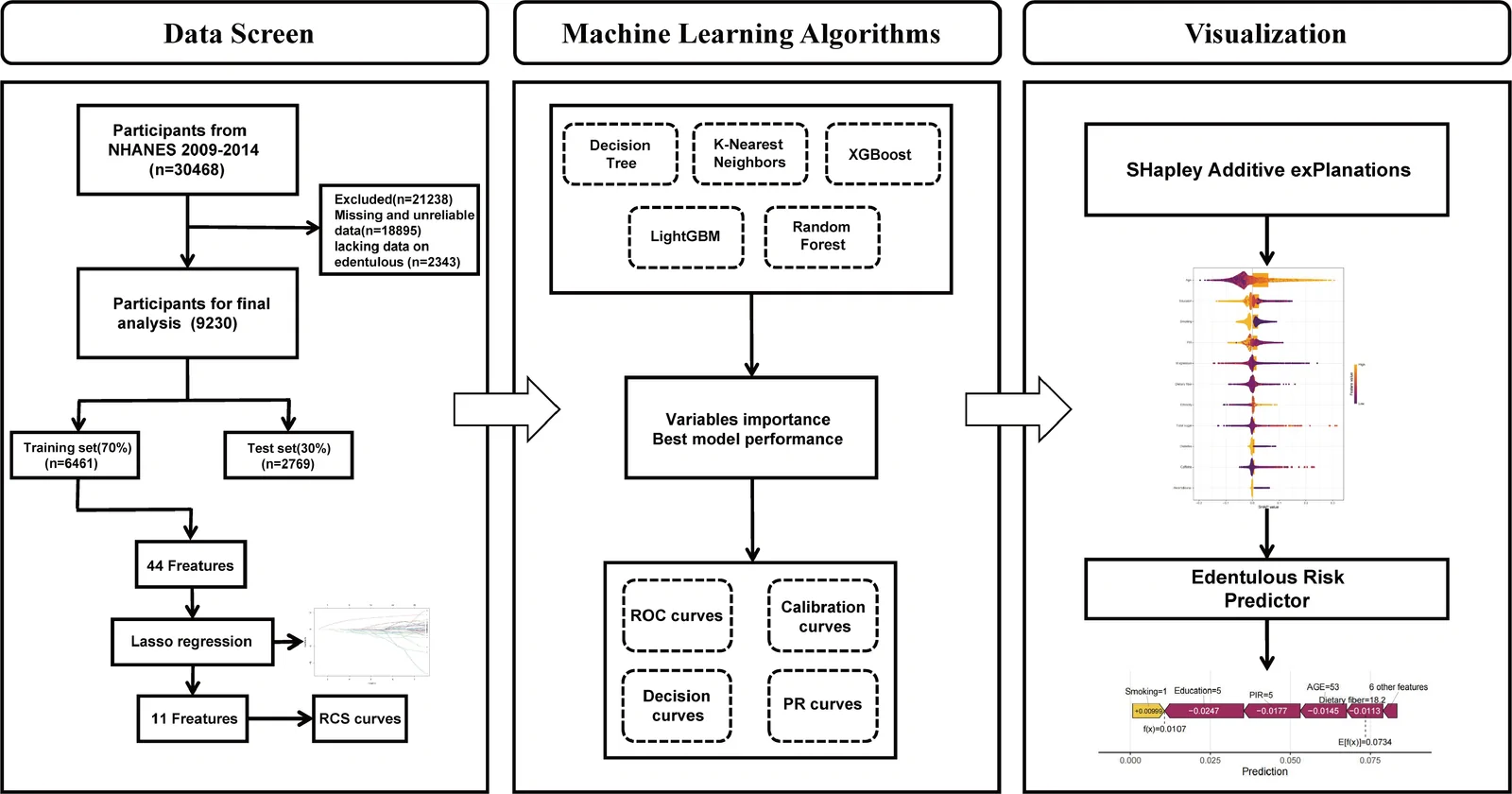
Study flow and interpretable machine learning framework for edentulism risk prediction. This figure illustrates the retrospective cross-sectional study design and the multistage machine learning framework used to develop and validate the edentulism risk prediction model in US adults. LASSO = least absolute shrinkage and selection operator, LightGBM = light gradient boosting machine, NHANES = National Health and Nutrition Examination Survey, PR = precision–recall, RCS = restricted cubic spline, ROC = receiver operating characteristic, XGBoost = extreme gradient boosting.

### 2.2. Definition and measurement of predictor variables

Predictors were selected based on prior evidence and clinical relevance and were categorized into 3 domains: demographic and socioeconomic factors, chronic disease/health indicators, and dietary exposures.

Demographic and socioeconomic variables included 8 factors – age, sex, race/ethnicity, educational attainment, poverty–income ratio (PIR), body mass index (BMI), marital status, and smoking status. Chronic disease indicators comprised 2 self-reported physician-diagnosed conditions: diabetes and hypertension. Dietary variables were derived from 24-hour dietary recall interviews and included intakes of vitamins (e.g., vitamins A, E, C), minerals (e.g., zinc, magnesium), and macronutrient-related components (e.g., total sugar, dietary fiber, caffeine). A full list of all predictors and their baseline distributions by outcome group is provided in Table 1.

**Table 1:** Baseline characteristics of participants included in the analysis (N = 9230).

	Total	No Edentulous	Edentulous	*P*-value
	(N = 9230)	(N = 8514)	(N = 726)	
Age, mean ± SD	53.55 ± 14.76	52.39 ± 14.41	67.35 ± 11.53	<.001
PIR, mean ± SD	2.64 ± 1.65	2.71 ± 1.66	1.84 ± 1.31	<.001
BMI, mean ± SD	29.49 ± 6.91	29.51 ± 6.93	29.29 ± 6.72	.428
Vitamin A, mean ± SD	638.52 ± 580.55	643.70 ± 586.49	577.00 ± 500.81	.003
Vitamin E, mean ± SD	8.26 ± 5.37	8.39 ± 5.44	6.70 ± 4.08	<.001
Vitamin C, mean ± SD	85.30 ± 78.26	86.58 ± 77.84	70.02 ± 81.58	<.001
Zinc, mean ± SD	11.12 ± 5.80	11.22 ± 5.76	9.92 ± 6.12	<.001
Selenium, mean ± SD	112.62 ± 50.97	113.73 ± 50.88	99.39 ± 50.19	<.001
Protein, mean ± SD	80.87 ± 34.01	81.76 ± 33.89	70.33 ± 33.72	<.001
Carbohydrate, mean ± SD	248.69 ± 102.19	250.42 ± 101.26	228.15 ± 110.68	<.001
Total sugar, mean ± SD	108.44 ± 62.86	108.63 ± 61.90	106.13 ± 73.32	.374
Dietary fiber, mean ± SD	17.51 ± 9.33	17.78 ± 9.36	14.36 ± 8.36	<.001
Total fat, mean ± SD	76.63 ± 36.88	77.32 ± 36.87	68.42 ± 36.12	<.001
Total saturated fatty acids, mean ± SD	24.60 ± 13.16	24.76 ± 13.14	22.72 ± 13.29	<.001
Total monounsaturated fatty acids, mean ± SD	27.37 ± 14.02	27.64 ± 14.04	24.12 ± 13.30	<.001
Cholesterol, mean ± SD	284.56 ± 183.24	286.28 ± 183.21	264.06 ± 182.39	.002
Retinol, mean ± SD	416.13 ± 421.27	415.98 ± 422.99	417.88 ± 400.51	.908
Thiamine, mean ± SD	1.60 ± 0.76	1.61 ± 0.76	1.43 ± 0.68	<.001
Riboflavin, mean ± SD	2.06 ± 1.04	2.08 ± 1.04	1.93 ± 1.11	<.001
Nicotinic acid, mean ± SD	25.05 ± 12.14	25.31 ± 12.02	22.07 ± 13.07	<.001
VitaminB6, mean ± SD	2.09 ± 1.26	2.11 ± 1.25	1.82 ± 1.27	<.001
Folic acid, mean ± SD	180.08 ± 147.24	181.95 ± 148.30	157.91 ± 132.13	<.001
Total choline, mean ± SD	332.83 ± 158.31	335.39 ± 157.86	302.31 ± 160.63	<.001
Vitamin B12, mean ± SD	4.97 ± 4.60	4.99 ± 4.59	4.77 ± 4.75	.24
Vitamin D, mean ± SD	4.85 ± 4.52	4.86 ± 4.55	4.70 ± 4.21	.35
Vitamin K, mean ± SD	119.30 ± 245.04	121.35 ± 248.49	94.90 ± 198.03	.006
Calcium, mean ± SD	932.42 ± 487.01	940.12 ± 486.57	840.88 ± 483.13	<.001
Phosphorus, mean ± SD	1352.02 ± 551.59	1366.58 ± 551.09	1178.83 ± 527.84	<.001
Magnesium, mean ± SD	300.06 ± 129.98	304.24 ± 129.70	250.38 ± 122.79	<.001
Copper, mean ± SD	1.27 ± 0.81	1.29 ± 0.81	1.08 ± 0.74	<.001
Sodium, mean ± SD	3421.86 ± 1445.81	3451.86 ± 1446.98	3065.18 ± 1383.82	<.001
Potassium, mean ± SD	2689.00 ± 1086.31	2713.03 ± 1076.02	2403.21 ± 1165.31	<.001
Caffeine, mean ± SD	155.38 ± 176.68	153.76 ± 173.23	174.63 ± 212.62	.011
Theobromine, mean ± SD	34.38 ± 57.81	34.52 ± 57.23	32.75 ± 64.35	.434
Alcohol, mean ± SD	8.08 ± 20.39	8.36 ± 20.49	4.84 ± 18.97	<.001
Total carotenoids, mean ± SD	9595.13 ± 10750.78	9803.88 ± 10814.90	7112.88 ± 9622.24	<.001
Gender, n(%)				.791
Male	4468 (48.41)	4118 (48.37)	350 (48.88)	
Female	4762 (51.59)	4396 (51.63)	366 (51.12)	
Ethnicity, n (%)				<.001
Mexican American	1144 (12.39)	1104 (12.97)	40 (5.59)	
Other Hispanic	809 (8.76)	765 (8.99)	44 (6.15)	
Non-Hispanic White	4418 (47.87)	4008 (47.08)	410 (57.26)	
Non-Hispanic Black	1916 (20.76)	1742 (20.46)	174 (24.30)	
Other race	943 (10.22)	895 (10.51)	48 (6.70)	
Education, n(%)				<.001
Less than ninth grade	800 (8.67)	674 (7.92)	126 (17.60)	
9–11th grade (includes 12th grade with no diploma)	1256 (13.61)	1067 (12.53)	189 (26.40)	
High school graduate/GED or equivalent	2026 (21.95)	1824 (21.42)	202 (28.21)	
Some college or AA degree	2649 (28.70)	2490 (29.25)	159 (22.21)	
College graduate or above	2499 (27.07)	2459 (28.88)	40 (5.59)	
Marital, n (%)				<.001
Married	5366 (58.14)	5042 (59.22)	324 (45.25)	
Widowed	796 (8.62)	620 (7.28)	176 (24.58)	
Divorced	1186 (12.85)	1076 (12.64)	110 (15.36)	
Separated	329 (3.56)	306 (3.59)	23 (3.21)	
Never married	1008 (10.92)	947 (11.12)	61 (8.52)	
Living with partner	545 (5.90)	523 (6.14)	22 (3.07)	
Smoking, n (%)				<.001
Yes	4239 (45.93)	3742 (43.95)	497 (69.41)	
No	4991 (54.07)	4772 (56.05)	219 (30.59)	
Heart disease, n (%)				<.001
Yes	392 (4.25)	304 (3.57)	88 (12.29)	
No	8838 (95.75)	8210 (96.43)	628 (87.71)	
Diabetes, n (%)				<.001
Yes	1335 (14.46)	1118 (13.13)	217 (30.31)	
No	7895 (85.54)	7396 (86.87)	499 (69.69)	

This table presents the baseline characteristics of all 9230 participants included in the final analysis, stratified by edentulous versus non-edentulous status. Continuous variables are shown as mean ± SD, and categorical variables as count (%). Between-group differences were tested with Student’s *t* test or χ^2^ test. The edentulous group was significantly older, had lower socioeconomic status (lower PIR and education level), higher rates of smoking and chronic diseases (heart disease, diabetes), and lower intake of antioxidant nutrients and dietary fiber (all *P* < .001).

Continuous variables indicate one-way analysis of means. Categorical variables indicate Pearson’s chi-squared test.

BMI = body mass index, GED = general educational development, PIR = poverty-to-income ratio, SD = standard deviation.

### 2.3. Outcome definition

The study outcome was edentulism. Edentulous status was determined from the NHANES oral health examination conducted by trained dental examiners. Participants with zero natural teeth recorded during the dental evaluation were classified as edentulous and treated as positive outcome events in all analyses.

### 2.4. Collection of baseline characteristics

Baseline characteristics were obtained from NHANES questionnaires and physical examinations. Age and PIR were treated as continuous variables, whereas sex, race/ethnicity, education, and marital status were categorized according to NHANES coding criteria. BMI was measured during physical examination.

Smoking status was classified as never smoker (lifetime cigarette consumption < 100 cigarettes) or smoker (≥100 cigarettes). Diabetes and cardiovascular disease histories were assessed through self-reported physician diagnoses (“Has a doctor ever told you that you have diabetes?”; “Has a doctor ever told you that you have heart disease, including coronary heart disease, angina, or myocardial infarction?”). All dietary indicators were extracted from 24-hour recall data and represent estimated daily nutrient intake.

### 2.5. Statistical analysis

All analyses were performed using R software (version 4.5.1; R Foundation for Statistical Computing) and relevant statistical and machine-learning packages (survey, tidyverse, DMwR, mlr3,caret, kernelshap, shapviz).

Continuous variables were summarized as mean ± standard deviation, and categorical variables as counts and percentages. Group differences were assessed using Student *t* test or χ^2^ test as appropriate. Missing data were handled via complete-case exclusion. The dataset was randomly split into training (70%) and testing (30%) subsets using stratified sampling to preserve outcome distribution.

Before model development, continuous predictors were scaled using Min–Max normalization, and categorical predictors were converted to one-hot encoded variables. Feature selection was conducted using least absolute shrinkage and selection operator (LASSO) logistic regression, with the penalization parameter λ determined by 20-fold cross-validation and the λ1SE (one standard error) criterion used to identify the final feature subset (Fig. 2). For key dietary predictors identified by LASSO, restricted cubic spline (RCS) models with 3 knots were used to characterize potential nonlinear dose–response relationships with edentulism.

**Figure 2.**
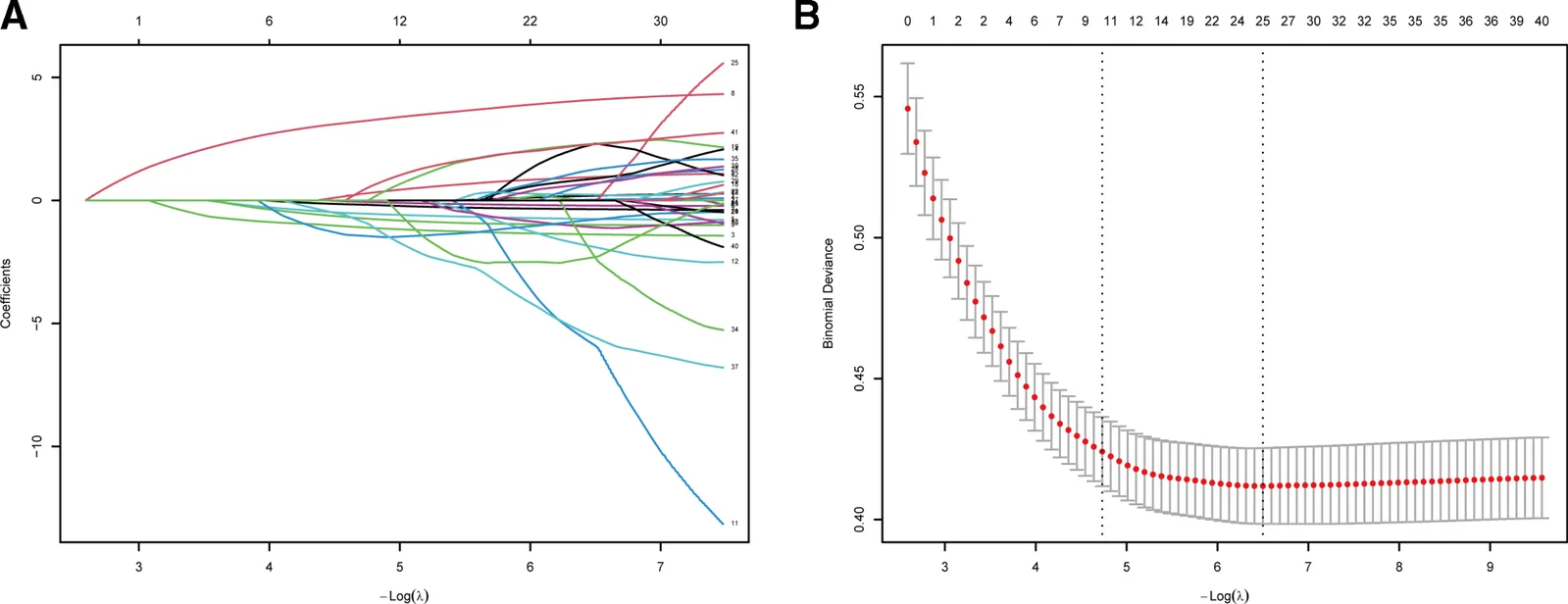
LASSO regression feature selection process. This figure depicts the regularization path for the LASSO (Least Absolute Shrinkage and Selection Operator) logistic regression model applied to the training set. The upper axis represents the number of nonzero coefficients, and the lower axis represents log(λ). The vertical dashed lines indicate the Min standard (the λ value corresponding to the minimum MSE) and the 1SE rule (the most regularized model within 1 standard error of the Min λ). Following the λ_1SE rule, 11 predictor variables were selected as the final core feature subset for downstream analysis. 1SE = one standard error, LASSO = least absolute shrinkage and selection operator, MSE = mean squared error.

Five machine-learning models – Random Forest, XGBoost, LightGBM, K-nearest neighbors, and Decision Tree – were trained using 10-fold cross-validation with grid-search tuning. Model performance in the testing set was assessed through discrimination (area under the receiver operating characteristic curve [AUC], area under the precision–recall curve [AUPRC]), calibration (calibration plots, expected calibration error), and clinical utility (decision curve analysis). Model interpretability was evaluated using SHAP values, including global importance plots (beeswarm), waterfall plots, and individual prediction explanations.

## 3. Result

### 3.1. Characteristics of the study population and data partitioning

A total of 9230 participants were included, among whom 726 (7.86%) were classified as edentulous (Table 1). Compared with non-edentulous individuals, edentulous participants were markedly older (67.35 ± 11.53 vs 52.39 ± 14.41 years, *P* < .001) and had substantially lower poverty–income ratios (1.84 ± 1.31 vs 2.71 ± 1.66, *P* < .001).

Dietary assessments revealed a consistent reduction in antioxidant vitamin and mineral intake among edentulous adults. Daily consumption of vitamins A, C, and E, as well as zinc, selenium, and magnesium, was significantly lower than in their non-edentulous counterparts (all *P* < .001). Similar patterns were observed for protein, dietary fiber, calcium, phosphorus, potassium, total fat (including monounsaturated and saturated fatty acids), and cholesterol (all *P* < .001).

Edentulous individuals also exhibited a markedly higher burden of cardiometabolic conditions. The prevalence of heart disease (12.29% vs 3.57%, *P* < .001) and diabetes (30.31% vs 13.13%, *P* < .001) was substantially elevated, and smoking was far more common (69.41% vs 43.95%, *P* < .001). Educational attainment further highlighted socioeconomic disparities: 44% of the edentulous group had less than a high-school education, while only 5.59% had completed college or above (*P* < .001).

Sex distribution did not differ significantly (*P* = .791). Across racial/ethnic strata, non-Hispanic White participants accounted for the largest proportion of edentulous cases (57.26%), whereas the prevalence was lower among Mexican American and other Hispanic subgroups (both *P* < .001). Collectively, these findings depict a characteristic profile of edentulous adults – older age, lower socioeconomic status, reduced antioxidant nutrient intake, and clustering of chronic diseases – providing a coherent epidemiological foundation for subsequent model development.

To reduce the risk of overfitting and ensure robust internal validation, this study applied stratified sampling to divide 9230 participants into a training set (n = 6461) and an internal test set (n = 2769) at a 7:3 ratio, using “edentulous status” as the stratification variable. As shown in Table 2, the 2 sets exhibited no significant differences in more than 95% of variables, indicating that stratified sampling effectively minimized population drift. The prevalence of edentulism was 7.85% in the training set and 7.15% in the test set (*P* = .627), and the sex distribution was identical in both groups (48.4% male vs 48.4% male, *P* = .212). Key continuous variables – including age, PIR, and BMI – also showed no statistical differences (all *P* > .05). Although vitamin A, vitamin C, and carbohydrate levels displayed slightly higher P-values (>0.20) in the test set, their directional trends remained consistent with those in the training set and can be considered random fluctuations.

**Table 2:** Comparison of baseline feature distributions between the training and test sets.

Characteristic	Cohort (training cohort), N = 6461			Cohort (internal test cohort), N = 2769		
	No edentulous	Edentulous	*P*-value*	No edentulous	Edentulous	*P*-value*
	N = 5954	N = 507		N = 2560	N = 198	
Age, mean ± SD	52.5 ± 14.4	67.6 ± 11.5	<.001	52.1 ± 14.5	66.9 ± 11.7	<.001
PIR, mean ± SD	2.7 ± 1.7	1.9 ± 1.3	<.001	2.7 ± 1.7	1.8 ± 1.3	<.001
BMI, mean ± SD	29.5 ± 6.9	29.2 ± 6.8	.349	29.5 ± 6.9	29.4 ± 6.7	.966
Vitamin A, mean ± SD	643.9 ± 553.3	572.8 ± 450.3	<.001	643.1 ± 657.4	587.2 ± 607.2	.205
Vitamin E, mean ± SD	8.4 ± 5.4	6.7 ± 4.0	<.001	8.4 ± 5.6	6.8 ± 4.4	<.001
Vitamin C, mean ± SD	86.1 ± 74.9	66.1 ± 64.4	<.001	87.6 ± 84.3	79.6 ± 112.6	.314
Zinc, mean ± SD	11.3 ± 5.6	10.0 ± 6.3	<.001	11.1 ± 6.1	9.7 ± 5.8	<.001
Selenium, mean ± SD	113.5 ± 50.3	100.3 ± 52.1	<.001	114.3 ± 52.3	97.2 ± 45.3	<.001
Total energy intake, mean ± SD	2056.9 ± 775.0	1813.0 ± 810.6	<.001	2051.7 ± 811.1	1841.1 ± 860.0	<.001
Protein, mean ± SD	81.9 ± 33.8	71.1 ± 34.7	<.001	81.5 ± 34.1	68.4 ± 31.2	<.001
Carbohydrate, mean ± SD	250.4 ± 99.8	224.8 ± 105.1	<.001	250.4 ± 104.7	236.3 ± 123.0	.11
Total sugar, mean ± SD	108.8 ± 61.3	103.2 ± 68.2	.073	108.2 ± 63.2	113.3 ± 84.2	.399
Dietary fiber, mean ± SD	17.7 ± 9.2	14.5 ± 8.4	<.001	17.9 ± 9.8	14.0 ± 8.2	<.001
Total fat, mean ± SD	77.4 ± 36.6	68.7 ± 36.1	<.001	77.1 ± 37.5	67.7 ± 36.2	<.001
Total saturated fatty acids, mean ± SD	24.8 ± 13.1	22.8 ± 13.4	.001	24.7 ± 13.2	22.6 ± 13.1	.024
Total monounsaturated fatty acids, mean ± SD	27.7 ± 13.9	24.3 ± 13.4	<.001	27.5 ± 14.3	23.7 ± 13.2	<.001
Cholesterol, mean ± SD	286.4 ± 182.0	267.5 ± 187.8	.03	286.1 ± 186.1	255.8 ± 168.6	.014
Retinol, mean ± SD	417.2 ± 446.4	411.3 ± 369.6	.736	413.1 ± 362.8	433.8 ± 467.8	.534
Thiamine, mean ± SD	1.6 ± 0.8	1.4 ± 0.7	<.001	1.6 ± 0.8	1.4 ± 0.6	<.001
Riboflavin, mean ± SD	2.1 ± 1.0	2.0 ± 1.2	.015	2.1 ± 1.0	1.9 ± 1.0	.01
Nicotinic.acid, mean ± SD	25.3 ± 11.9	22.2 ± 13.7	<.001	25.2 ± 12.3	21.9 ± 11.4	<.001
Vitamin B6, mean ± SD	2.1 ± 1.2	1.8 ± 1.3	<.001	2.1 ± 1.3	1.8 ± 1.3	.003
Folic acid, mean ± SD	183.5 ± 152.3	158.8 ± 135.3	<.001	178.4 ± 138.6	155.8 ± 124.2	.013
Total choline, mean ± SD	335.7 ± 156.3	306.2 ± 165.2	<.001	334.7 ± 161.4	292.8 ± 148.9	<.001
Vitamin B12, mean ± SD	5.0 ± 4.8	4.8 ± 4.6	.232	4.9 ± 4.1	4.8 ± 5.2	.767
Vitamin D, mean ± SD	4.9 ± 4.5	4.8 ± 4.4	.659	4.9 ± 4.7	4.5 ± 3.7	.206
Vitamin K, mean ± SD	119.3 ± 133.1	89.0 ± 117.2	<.001	126.2 ± 405.2	109.1 ± 318.0	.466
Calcium, mean ± SD	940.6 ± 482.6	834.2 ± 470.6	<.001	939.0 ± 495.7	857.1 ± 513.1	.027
Phosphorus, mean ± SD	1368.0 ± 546.2	1185.0 ± 532.3	<.001	1363.3 ± 562.3	1163.9 ± 517.9	<.001
Magnesium, mean ± SD	304.7 ± 127.6	249.1 ± 108.4	<.001	303.1 ± 134.6	253.4 ± 152.4	<.001
Copper, mean ± SD	1.3 ± 0.8	1.1 ± 0.6	<.001	1.3 ± 0.7	1.1 ± 0.9	.033
Sodium, mean ± SD	3450.8 ± 1435.1	3065.7 ± 1394.4	<.001	3454.3 ± 1474.5	3063.9 ± 1361.2	<.001
Potassium, mean ± SD	2716.3 ± 1034.5	2407.5 ± 1005.1	<.001	2705.4 ± 1167.2	2392.7 ± 1486.6	.003
Caffeine, mean ± SD	154.8 ± 172.6	183.2 ± 228.4	.006	151.4 ± 174.7	153.9 ± 167.0	.836
Theobromine, mean ± SD	34.5 ± 57.7	34.7 ± 70.3	.957	34.5 ± 56.2	28.0 ± 46.7	.06
Alcohol, mean ± SD	8.4 ± 20.8	4.7 ± 18.0	<.001	8.3 ± 19.8	5.3 ± 21.1	.046
Total.carotenoids, mean ± SD	9721.6 ± 9551.2	6924.5 ± 7693.3	<.001	9995.3 ± 13297.6	7569.8 ± 13190.6	.011
Gender, n (%)			.627			.212
Male	2880 (48.4%)	239 (47.1%)		1238 (48.4%)	111 (53.1%)	
Female	3074 (51.6%)	268 (52.9%)		1322 (51.6%)	98 (46.9%)	
Ethnicity, n (%)			<.001			<.001
Mexican American	756 (12.7%)	35 (6.9%)		348 (13.6%)	5 (2.4%)	
Other Hispanic	506 (8.5%)	27 (5.3%)		259 (10.1%)	17 (8.1%)	
Non-Hispanic White	2830 (47.5%)	290 (57.2%)		1178 (46.0%)	120 (57.4%)	
Non-Hispanic Black	1252 (21.0%)	118 (23.3%)		490 (19.1%)	56 (26.8%)	
Other race	610 (10.2%)	37 (7.3%)		285 (11.1%)	11 (5.3%)	
Education, n (%)			<.001			<.001
Less than ninth grade	462 (7.8%)	93 (18.3%)		212 (8.3%)	33 (15.8%)	
9–11th grade (includes 12th grade with no diploma)	727 (12.2%)	144 (28.4%)		340 (13.3%)	45 (21.5%)	
High school graduate/GED or equivalent	1294 (21.7%)	132 (26.0%)		530 (20.7%)	70 (33.5%)	
Some college or AA degree	1744 (29.3%)	112 (22.1%)		746 (29.1%)	47 (22.5%)	
College graduate or above	1727 (29.0%)	26 (5.1%)		732 (28.6%)	14 (6.7%)	
Marital, n (%)			<.001			<.001
Married	3497 (58.7%)	230 (45.4%)		1545 (60.4%)	94 (45.0%)	
Widowed	435 (7.3%)	130 (25.6%)		185 (7.2%)	46 (22.0%)	
Divorced	785 (13.2%)	78 (15.4%)		291 (11.4%)	32 (15.3%)	
Separated	214 (3.6%)	15 (3.0%)		92 (3.6%)	8 (3.8%)	
Never married	662 (11.1%)	40 (7.9%)		285 (11.1%)	21 (10.0%)	
Living with partner	361 (6.1%)	14 (2.8%)		162 (6.3%)	8 (3.8%)	
Smoking, n (%)			<.001			<.001
Yes	2635 (44.3%)	358 (70.6%)		1107 (43.2%)	139 (66.5%)	
No	3319 (55.7%)	149 (29.4%)		1453 (56.8%)	70 (33.5%)	
Heart disease, n (%)			<.001			<.001
Yes	214 (3.6%)	67 (13.2%)		90 (3.5%)	21 (10.0%)	
No	5740 (96.4%)	440 (86.8%)		2470 (96.5%)	188 (90.0%)	
Diabetes, n (%)			<.001			<.001
Yes	778 (13.1%)	161 (31.8%)		340 (13.3%)	56 (26.8%)	
No	5176 (86.9%)	346 (68.2%)		2220 (86.7%)	153 (73.2%)	

This table compares the distribution of all predictor variables between the training set (n = 6461) and the internal test set (n = 2769) after stratified random sampling by edentulous status. The comparison ensures that the 2 cohorts are homogeneous, thereby enabling an unbiased performance evaluation of the machine-learning models. The distributions of the vast majority of core variables – including edentulism status, age, PIR, BMI and chronic disease history – showed no statistically significant differences between the 2 sets (*P* > .05).

BMI = body mass index, GED = general educational development, PIR = poverty-to-income ratio, SD = standard deviation.

* Pearson’s Chi-squared test; Welch two sample *t* test.

Notably, antioxidant-related features (vitamin E, zinc, selenium, magnesium, dietary fiber, and total carotenoids) demonstrated highly consistent distribution gradients between the training and test sets (all *P* > .05), ensuring the stability of dietary exposure distributions for subsequent model development. Chronic disease–related variables also showed nearly overlapping proportions, including diabetes (13.1% vs 13.3%), heart disease (3.6% vs 3.5%), and smoking status (44.3% vs 43.2%; all *P* > .05). Sociodemographic factors such as education level, marital status, and race likewise exhibited no systematic differences.

In summary, Table 2 clearly demonstrates that the stratified random sampling strategy successfully maintained homogeneity between the training and test sets across the edentulous–non-edentulous spectrum, thereby providing a solid foundation for unbiased and reproducible internal validation of the subsequent machine-learning models.

### 3.2. Feature selection and nonlinear relationship analysis

Following the initial LASSO screening, 11 predictors were ultimately retained, including age, race, education, PIR, smoking status, magnesium, dietary fiber, total sugar intake, caffeine, diabetes, and heart disease (Fig. 2). For the 4 continuous dietary variables retained – total sugar, dietary fiber, magnesium, and caffeine – we further applied restricted cubic splines (RCS) to examine the dose–response patterns associated with edentulism risk.

Figure 3A shows a significant nonlinear positive association between total sugar intake and edentulism risk (*P*-overall < .001; *P*-nonlinear < .001). When daily total sugar intake is <100 g, the risk curve declines slowly. Beyond this threshold, the curve turns upward, though with a relatively gentle slope – each 50 g increase is associated with approximately a 1.8% increase in the probability of edentulism (95% confidence interval: 1.2–2.4%). In contrast with total sugar, dietary fiber exhibits a pronounced nonlinear protective effect in Figure 3B (*P*-overall < .001; *P*-nonlinear < .001). When intake ranges from 0 to 20 g/d, the risk decreases rapidly with higher intake; beyond 20 g/d, the curve plateaus, indicating a “basic-dose” effect.

**Figure 3.**
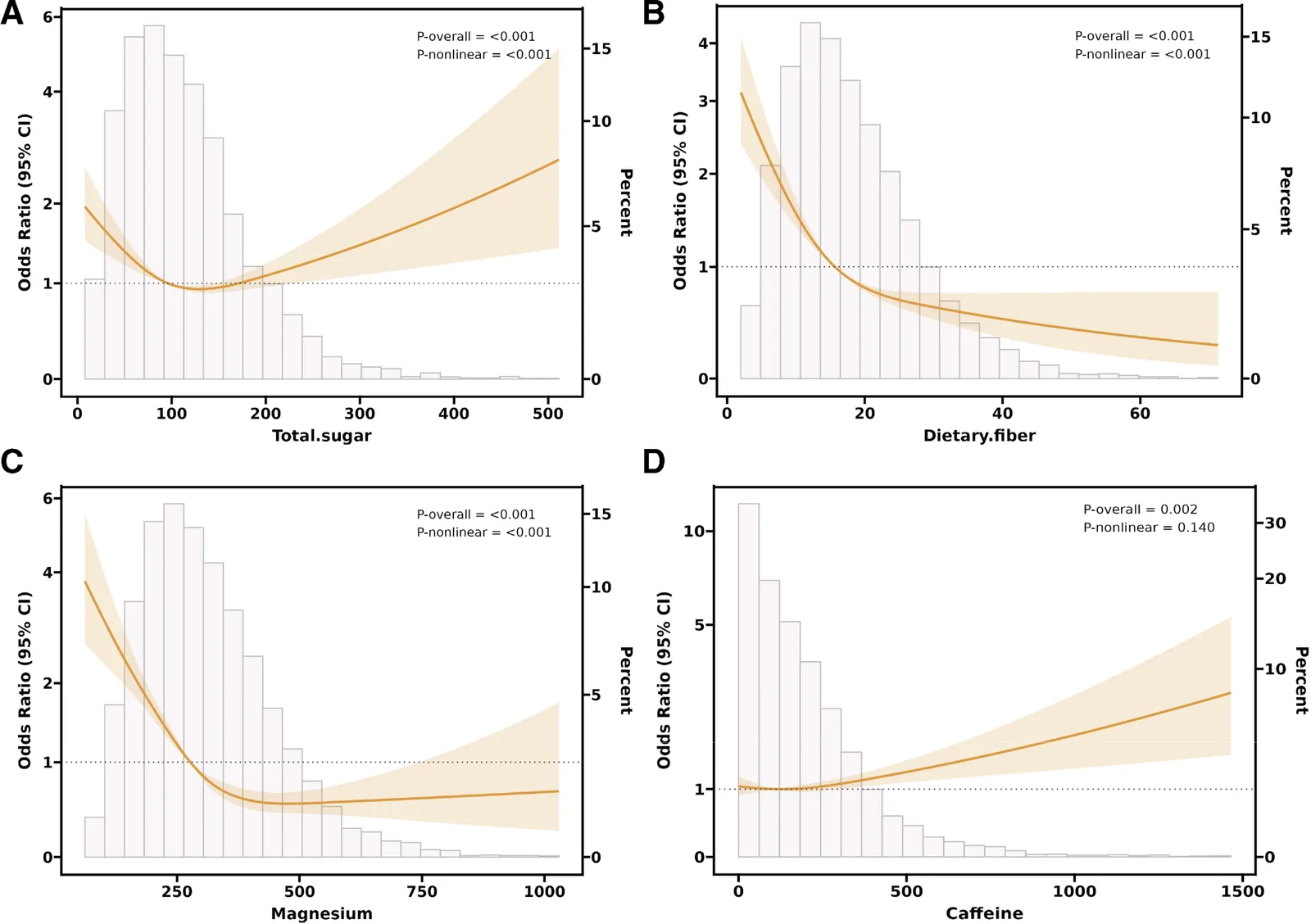
Restricted cubic spline curves for the dose–response association between key dietary factors and edentulism risk. Restricted cubic spline (RCS) regression (3 knots) was applied to characterize potential nonlinear relations of 4 continuous dietary variables with incident edentulism. The solid orange line denotes the odds ratio (OR); the shaded band indicates the 95% confidence interval (CI). The horizontal dashed line (OR = 1) is the reference. Histogram bars show the percentage distribution of participants across intake levels. (A) Total sugar intake and edentulism risk; (B) dietary fiber intake and edentulism risk; (C) magnesium intake and edentulism risk; (D) caffeine intake and edentulism risk. CI = confidence interval, OR = odds ratio, RCS = restricted cubic spline.

Magnesium intake similarly demonstrates a significant nonlinear protective pattern (Fig. 3C; *P*-overall < .001; *P*-nonlinear < .001). When magnesium intake is <250 mg/d, edentulism risk declines in an approximately linear manner; above 250 mg/d, the slope becomes markedly flatter, suggesting that 250 mg may serve as a risk-stratification cut point. As shown in Figure 3D, caffeine intake is mildly positively associated with edentulism risk (*P*-overall = .002; *P*-nonlinear = .140). The curve remains nearly flat between 0 to 200 mg/d, increasing slowly thereafter without a clear inflection point, reflecting an overall “plateau-to-gentle-rise” linear trend.

### 3.3. Development and validation of the edentulism prediction model

Table 3 summarizes the comprehensive performance of the 5 algorithms in the internal test set (n = 2769). As shown in the receiver operating characteristic (ROC) curves in Figure 4, LightGBM and XGBoost tied for the best performance (AUC = 0.855), followed closely by Random Forest (0.842), which substantially outperformed Decision Tree (0.761) and K-nearest neighbors (KNN; 0.587). In the precision–recall curves under the imbalanced outcome distribution (positive rate = 7.2%), gradient boosting methods again demonstrated clear superiority: the curves of LightGBM and XGBoost remained the outermost across the full range, with precision declining the slowest as recall increased from 0.2 to 0.8. Random Forest ranked slightly behind but remained clearly superior to Decision Tree, whereas the KNN curve was the most concave, showing the lowest precision within the same recall intervals.

**Table 3:** Comparison of performance metrics for 5 prediction models on the test set.

Metrics of the 5 machine learning models in predicting edentulous					
Machine learner	Acurracy	Area under the ROC curve	Sensitivity	Specificity	ECE
Decition Tree	0.815	0.761	0.56	0.836	0.0221
LightGBM	0.808	0.855	0.656	0.82	0.0154
XGboost	0.81	0.855	0.675	0.821	0.0128
KNN	0.889	0.587	0.23	0.943	0.108
Random Forest	0.809	0.842	0.679	0.819	0.0097

This table summarizes the key performance indicators for the 5 developed machine-learning algorithms (Random Forest, XGBoost, LightGBM, Decision Tree and K-Nearest Neighbors) evaluated on the independent internal test set. Metrics include discrimination (AUC and AUPRC), reliability (expected calibration error, ECE) and classification accuracy (sensitivity and specificity). The model demonstrating the best overall balance of discrimination, reliability and clinical utility was selected as the final prediction model.

AUC = area under the receiver operating characteristic curve, AUPRC = area under the precision–recall curve, ECE = expected calibration error, KNN = K-nearest neighbors, LightGBM = light gradient boosting machine, ROC = receiver operating characteristic, XGBoost = extreme gradient boosting.

**Figure 4.**
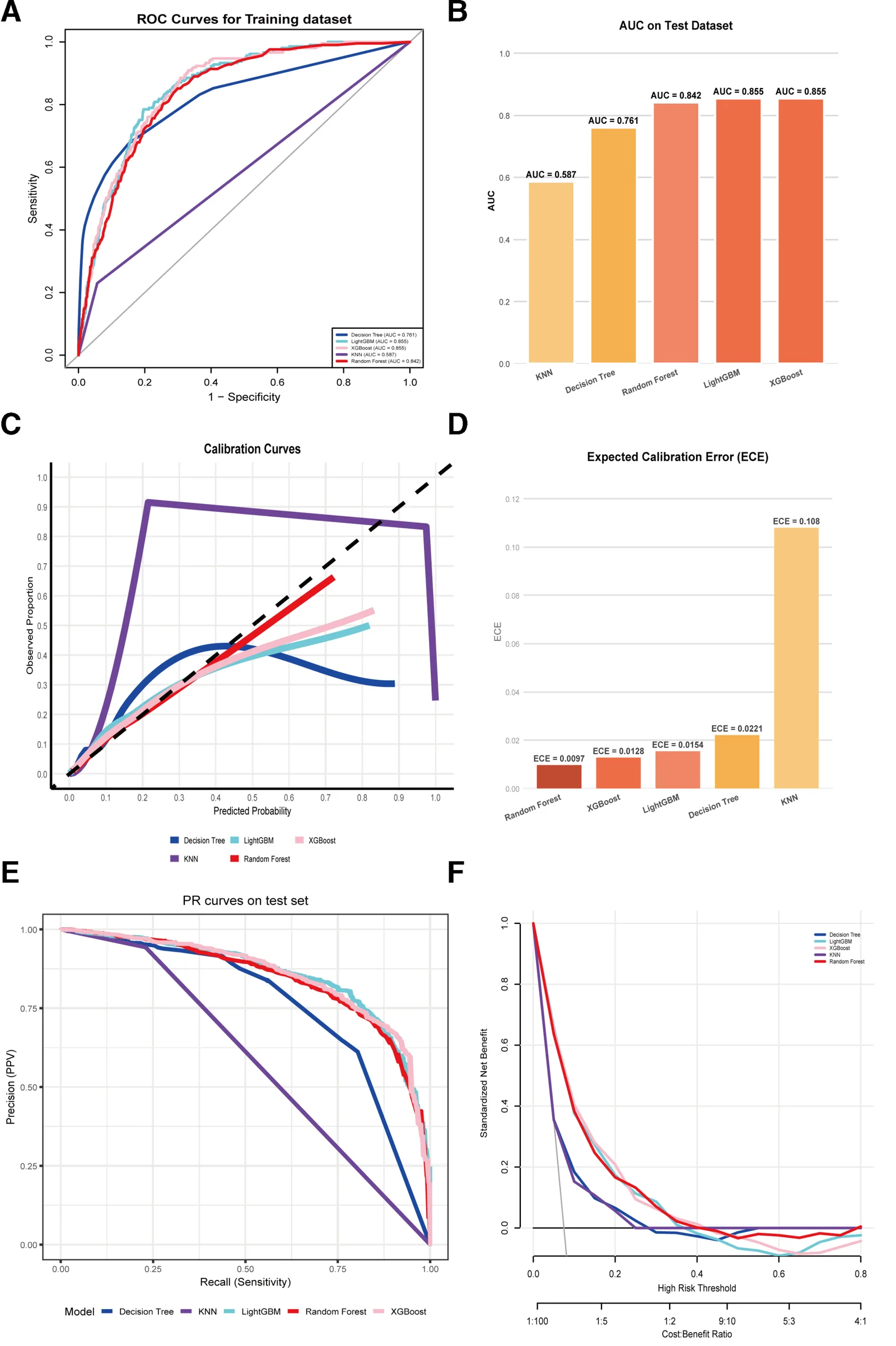
Performance of the 5 prediction models on the internal test set (n = 2769). Models: Random Forest (RF), XGBoost, LightGBM, Decision Tree (DT) and K-Nearest Neighbours (KNN). (A) Receiver-operating characteristic (ROC) curves. (B) Area under the ROC curve (AUC). (C) Calibration plots. (D) Expected calibration error (ECE). (E) Precision–recall curves. (F) Decision-curve analysis. AUC = area under the receiver operating characteristic curve, DT = Decision Tree, ECE = expected calibration error, KNN = K-nearest neighbors, LightGBM = light gradient boosting machine, ROC = receiver operating characteristic, XGBoost = extreme gradient boosting.

Regarding sensitivity, Random Forest achieved 0.679, outperforming LightGBM (0.656) and Decision Tree (0.560), while maintaining a specificity of 0.819 and thus achieving a favorable balance between recall and precision. In terms of calibration, the predicted probabilities of Random Forest closely aligned with the diagonal, with an expected calibration error (ECE) of only 0.0097 – the lowest among all models. LightGBM (0.0154) and XGBoost (0.0128) also showed <2% calibration error, whereas Decision Tree (0.0221) and especially KNN (0.108) exhibited considerable degradation, with KNN systematically overestimating risk in the 0.4 to 0.6 probability range.

Decision curve analysis revealed that when the high-risk threshold was set between 35% and 80% (corresponding to a cost–benefit ratio of approximately 1:2.3 to 4:1), Random Forest yielded a markedly higher net benefit than both XGBoost and LightGBM across most threshold points, and consistently outperformed the extreme strategies of “intervene for all” and “intervene for none.” Beyond 80%, the net benefit gap narrowed rapidly and clinical usefulness diminished accordingly. Considering AUC, precision–recall (PR) curves, the sensitivity–specificity balance, ECE, and decision curve analysis [DCA] collectively, Random Forest achieved the most favorable trade-off between discrimination, probability reliability, and clinical net benefit. Therefore, it was selected as the final prediction model and subsequently subjected to SHAP-based interpretability analysis.

### 3.4. Interpretability of the final prediction model

The SHAP summary plot (Fig. 5A), ranked by mean absolute SHAP values, identified age, years of education, smoking status, PIR, magnesium, and dietary fiber as the top contributors to edentulism risk. High age values clustered on the right side, indicating that advanced age markedly increases predicted risk. In contrast, higher education, nonsmoking status, and higher PIR primarily appeared on the left side, suggesting strong negative contributions to risk. The SHAP value distributions of magnesium and dietary fiber were roughly symmetrical, indicating that their protective effects were consistent across varying intake levels.

**Figure 5.**
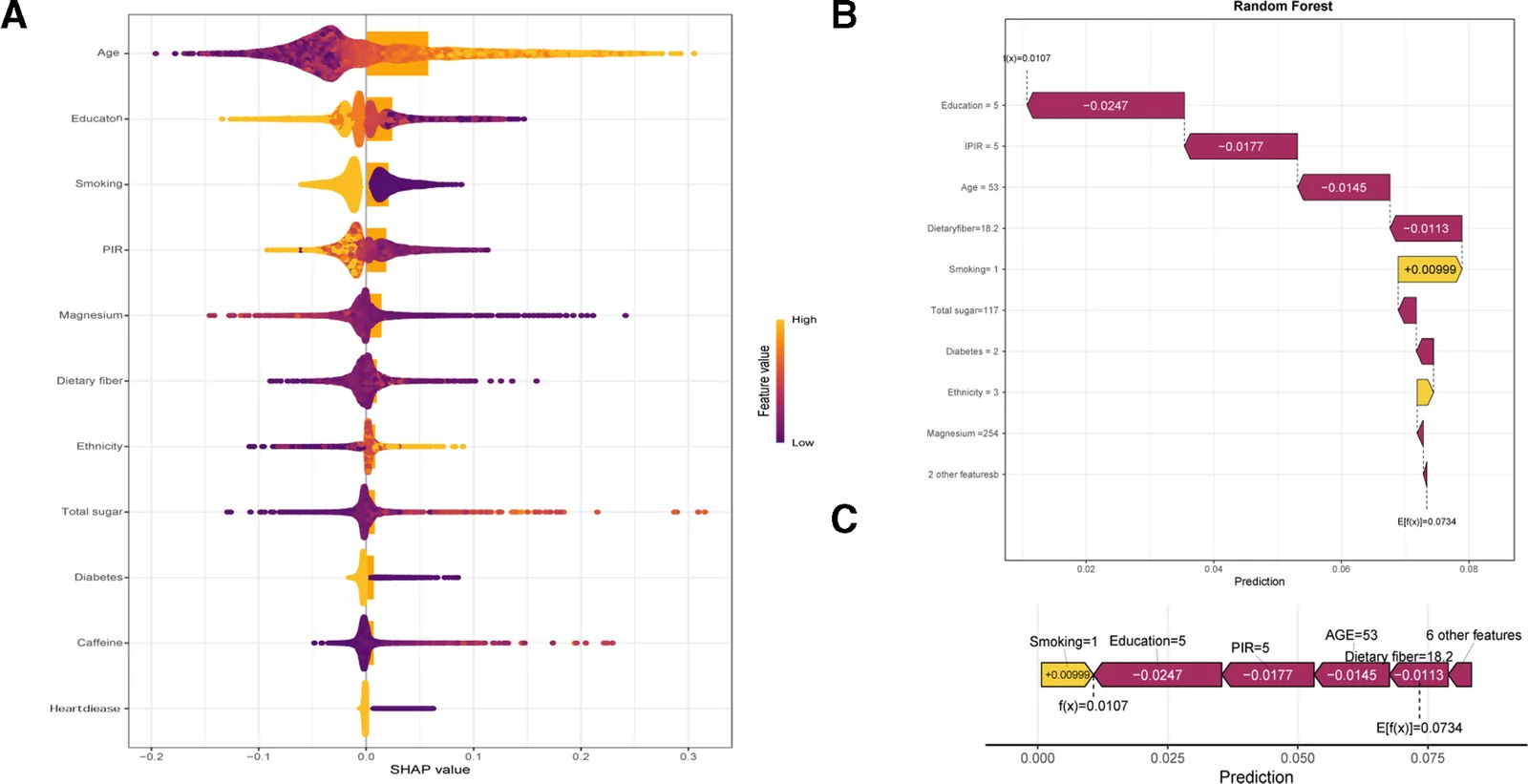
SHAP-based interpretability of the final Random Forest model. (A) Global feature importance (beeswarm): Features are ranked by mean |SHAP value|. Each point is 1 participant; color indicates high (red) or low (blue) feature value. Age, education, smoking and PIR exert the strongest influence on predicted edentulism probability. (B) Local explanation for a single participant (waterfall): Starting from the model base value (0.098), bars show the incremental SHAP contribution of each variable. Education = 5 decreases risk most, whereas Smoking = 1 increases risk; the final predicted probability is 0.0734. (C) Local explanation for the same participant (force plot): Positive SHAP values (red) push the log-odds rightward (higher risk); negative values (blue) pull leftward (lower risk). The net effect yields *f*(*x*) = 0.0107 log-odds, corresponding to a probability of .0734. PIR = poverty–income ratio, SHAP = SHapley Additive exPlanations.

Figure 5B presents a waterfall plot for an individual with a predicted probability of 0.073. The baseline risk of 0.098 decreased to 0.073 after adjusting for individual predictors. Higher educational attainment (Education = 5) contributed the largest protective effect (–0.0247), followed by high PIR (–0.0177), age 53 years (–0.0145), and dietary fiber intake of 18.2 g/d (–0.0113). Smoking was the only factor exerting a positive influence (+0.00999), whereas contributions from other variables were nearly negligible. The single-sample force plot (Fig. 5C) further visualized these effects: bars on the right collectively pulled the predicted value downward from the baseline, while the single bar on the left (Smoking = 1) exerted a mild upward pull, yielding a final prediction of 0.0107.

## 4. Discussion

Leveraging a nationally representative US sample, this study innovatively integrated 5 machine-learning algorithms with multidimensional health features to develop and validate an interpretable prediction model for edentulism. Our findings indicate that edentulism is not driven by a single factor but instead arises from complex nonlinear interactions among sociodemographic characteristics, behavioral factors, and dietary nutritional exposures. Among the 5 models, Random Forest (RF) was identified as the optimal algorithm, achieving the best overall balance of discrimination (AUC = 0.842), calibration accuracy (ECE = 0.0097), and clinical net benefit across a wide range of decision thresholds. Importantly, SHAP-based explanations not only quantified the contributions of traditional risk factors such as age, smoking, and socioeconomic status (SES), but – via restricted cubic spline (RCS) analyses – also revealed, for the first time, pronounced “L-shaped” nonlinear protective thresholds for dietary magnesium and fiber intake, offering explicit quantitative targets for precision nutrition interventions.

Previous edentulism prediction models have relied largely on logistic regression, which suffers from performance limitations in the presence of multicollinearity and high-dimensional feature interactions. By contrast, the ensemble-learning approach adopted here – particularly Random Forest and XGBoost – displayed clear methodological advantages. Although XGBoost and LightGBM slightly outperformed RF in discrimination (AUC = 0.855), RF demonstrated markedly superior calibration, a property that is essential for clinical risk assessment, where accurate probability estimation is often more valuable than binary classification alone. Furthermore, our SHAP analysis overcomes the typical “black-box” limitation of machine-learning models by visually illustrating how low educational attainment and smoking jointly elevate individual-level risk, thereby substantially improving the model’s transparency and acceptability in clinical settings.

A key finding of this study is that insufficient dietary magnesium intake emerged as a major predictor of edentulism, with RCS analyses indicating ~250 mg/d as a potential protective threshold. This result aligns closely with recent advances in the literature. Magnesium is essential for maintaining alveolar bone density and mineral crystallinity, and it also plays a central role in immunoinflammatory regulation.^[19-21]^ Molecular studies have shown that magnesium deficiency activates the nuclear factor kappa B (NF-κB) pathway and stimulates the excessive release of inflammatory mediators such as interleukin-1 beta (IL-1β) and tumor necrosis factor alpha (TNF-α), thereby exacerbating periodontal inflammation and accelerating alveolar bone resorption.^[22]^ Moreover, magnesium mitigates oxidative stress by reducing reactive oxygen species (ROS)–mediated damage to periodontal ligament cells.^[23]^ Our model further demonstrates that when intake falls below ~250 mg/d, the risk of edentulism rises steeply, suggesting that typical dietary intake may be insufficient for optimal bone protection in older adults and that magnesium supplementation could be a valuable preventive strategy.

Dietary fiber emerged as another major protective factor, displaying a clear saturation pattern, with risk reduction plateauing at intakes >20 g/d. This nonlinear association offers new insight for dietary guidelines. While earlier research emphasized mechanical stimulation through mastication as the primary benefit of fiber for periodontal health,^[24,25]^ recent microbiome studies propose a more systemic mechanism: dietary fiber functions as a prebiotic, modulating the gut microbiota and enhancing the production of short-chain fatty acids (SCFAs).^[26]^ SCFAs not only improve insulin sensitivity – thereby lowering the risk of diabetes, a major comorbidity of edentulism – but also act through the gut–oral axis to inhibit oral pathogen colonization, reducing the risk of periodontitis and tooth loss.^[27-29]^ Our findings support this systemic regulatory hypothesis and suggest that consuming at least 20 g/d of dietary fiber may confer maximal oral-health benefits among individuals at high risk.

The SHAP summary plot also reaffirmed age, educational attainment, and the poverty–income ratio (PIR) as the strongest drivers of edentulism risk. This aligns with the well-established “social gradient” in oral-health disparities: individuals with lower SES face a disproportionately high disease burden. Beyond limited access to dental care, low-SES groups are more frequently exposed to high-sugar diets and smoking – both of which contribute to tooth loss.^[30,31]^ Notably, smoking ranked immediately after age and education in SHAP contribution and showed a clear positive effect, underscoring that despite the importance of nutritional interventions, smoking cessation and broader socioeconomic support (e.g., expanding dental insurance coverage) remain essential components of edentulism prevention efforts.

This study has several strengths, including the use of nationally representative, high-quality data and the integration of advanced machine-learning algorithms with RCS analyses, which not only improved predictive performance but also quantified the dose–response relationships of key nutrients. Nonetheless, certain limitations must be acknowledged. First, the cross-sectional nature of NHANES precludes causal inference, and bidirectional associations are possible – such as individuals with edentulism reducing fiber intake due to chewing difficulties. Second, the 24-hour dietary recall is subject to recall bias and may not fully reflect long-term dietary patterns. Third, although we included multiple chronic conditions, the absence of detailed clinical indicators for periodontitis and dental caries may contribute to residual confounding. Future prospective cohort studies incorporating biochemical nutritional biomarkers are needed to validate the model’s external generalizability.

In conclusion, this study developed an interpretable Random Forest–based prediction model for edentulism that demonstrates high accuracy and strong clinical utility. Our findings highlight the multidimensional interaction among age, SES, and dietary antioxidant intake in shaping oral terminal health, and they identify potential protective thresholds for magnesium and dietary fiber. This model may serve as a valuable tool for community-based oral-health screening among older adults and provides scientific support for precision oral-disease prevention strategies centered on anti-inflammatory dietary interventions.

## 5. Conclusion

This study successfully developed and validated a high-accuracy, interpretable prediction model for edentulism risk. Using large-scale data from the US National Health and Nutrition Examination Survey (NHANES), we systematically compared 5 mainstream machine learning algorithms and identified the Random Forest model as the optimal approach. In an independent testing dataset, it demonstrated excellent discrimination (AUC = 0.842), accurate calibration, and robust net clinical benefit.

Through SHAP-based interpretability analysis, the model not only quantified the relative contributions of conventional risk factors – such as advanced age, low socioeconomic status, and smoking – but, importantly, incorporated dietary nutrients into a high-dimensional predictive framework for the first time. We identified inadequate intake of dietary magnesium and dietary fiber as important predictors of edentulism. Moreover, dose–response analyses revealed nonlinear associations between these key nutrients and edentulism risk, providing evidence-based thresholds (e.g., approximately 20 g/d of dietary fiber) that may support the development of precision nutrition–oriented intervention strategies.

## Acknowledgments

The authors express their gratitude to the participants and staff of the NHANES program for their longstanding contributions to national health surveillance. We also acknowledge the support provided by the open-access data infrastructure maintained by the National Center for Health Statistics, which made this research possible.

## Author contributions

**Conceptualization:** Shiyuan Wu, Yaw-Syan Fu.

**Data curation:** Shiyuan Wu, Muze Huang, Zi-han Shen, Yaw-Syan Fu, Rong Wang.

**Formal analysis:** Shiyuan Wu, Muze Huang, Zi-han Shen, Yaw-Syan Fu, Rong Wang.

**Funding acquisition:** Yaw-Syan Fu.

**Investigation:** Zi-han Shen, Yaw-Syan Fu, Rong Wang.

**Methodology:** Shiyuan Wu, Zi-han Shen, Yaw-Syan Fu.

**Project administration:** Shiyuan Wu, Yaw-Syan Fu, Rong Wang.

**Resources:** Shiyuan Wu, Yaw-Syan Fu.

**Software:** Shiyuan Wu.

**Supervision:** Rong Wang, Yaw-Syan Fu.

**Validation:** Shiyuan Wu, Yaw-Syan Fu, Rong Wang.

**Visualization:** Shiyuan Wu.

**Writing – original draft:** Shiyuan Wu, Muze Huang, Zi-han Shen.

**Writing – review & editing:** Shiyuan Wu, Yaw-Syan Fu, Rong Wang.
